# Outcome of an HIV education program for primary care providers: Screening and late diagnosis rates

**DOI:** 10.1371/journal.pone.0218380

**Published:** 2019-07-02

**Authors:** Javier Martínez Sanz, María Jesús Pérez Elías, Alfonso Muriel, Cristina Gómez Ayerbe, María Jesús Vivancos Gallego, Matilde Sánchez Conde, Margarita Herrero Delgado, Pilar Pérez Elías, Lidia Polo Benito, Yolanda de la Fuente Cortés, Rafael Barea, Ann K. Sullivan, Maria Jose Fuster Ruiz de Apodaca, María José Galindo, Santiago Moreno, for the DRIVE 03, OPTtest WP5 and FOCO Study Groups

**Affiliations:** 1 Department of Infectious Diseases, Hospital Universitario Ramón y Cajal, IRYCIS, Madrid, Spain; 2 Biostatistics Unit, Hospital Universitario Ramón y Cajal, IRYCIS, Madrid, Spain; 3 CIBER de Epidemiología y Salud Pública (CIBERESP), Madrid, Spain; 4 Centro de Salud Mar Báltico, Madrid, Spain; 5 Centro de Salud García Noblejas, Madrid, Spain; 6 Hospital General Universitario Gregorio Marañón, Madrid, Spain; 7 Centro de Salud Aquitania, Madrid, Spain; 8 Centro de Salud Canal de Panamá, Madrid, Spain; 9 Chelsea and Westminster Healthcare NHS Foundation Trust, London, United Kingdom; 10 Sociedad Española Interdisciplinaria del Sida (SEISIDA), Madrid, Spain; 11 Infectious Diseases Unit, Hospital Clínico Universitario de Valencia, Valencia, Spain; University of Ghana College of Health Sciences, GHANA

## Abstract

**Background:**

Late HIV diagnosis remains one of the challenges in combating the epidemic. Primary care providers play an important role in screening for HIV infection. Our study aims to evaluate the relationship between knowledge and barriers to HIV testing and screening outcomes. The impact of an education program for primary care providers, towards improving HIV testing and late diagnosis rates, is also assessed.

**Methods:**

A self-administered questionnaire that was developed within the framework of the European project OptTEST was used to examine HIV knowledge and barriers to HIV testing scores before and after being involved in an HIV education program. A quasi-experimental design with pre- and post-intervention measures was performed to investigate its impact. We performed multivariable logistic regression analysis to assess the relationship between variables for the HIV testing offer.

**Results:**

A total of 20 primary care centers and 454 primary care staff were included. Baseline OptTEST results showed that more knowledgeable staff offered an HIV test more frequently (OR 1.07; CI 95% 1.01–1.13; *p* = 0.027) and had lower barrier scores (OR 0.89; CI 95% 0.77–0.95; p = 0.005). Nurses had lower scores in knowledge-related items (OR 0.28; CI 95% 0.17–0.46; p<0.001), but higher scores in barrier-related items than physicians (OR 3.28; CI 95% 2.01–5.46; p<0.001). Specific centers with more knowledgeable staff members had a significant association with a greater level of new HIV diagnosis rates (OR 1.61; CI 95% 1.04–2.49; p = 0.032). After the intervention, we found that 12 out of 14 individual questions showed improved scores. In the 6 months after the training program, we similarly found a higher HIV testing rate (OR 1.19; CI 1.02–1.42; p = 0.036).

**Conclusions:**

This study highlights the association between knowledge and barriers to HIV testing, including HIV testing rates. It shows that it is possible to modify knowledge and reduce perceived barriers through educational programs, subsequently improving HIV screening outcomes.

## Introduction

Spain is one of the European countries with the highest estimated number of people living with HIV, and it still has a high rate of new HIV diagnoses [1,2]. Of an estimated 150,000 people living with HIV in Spain, 18% remain undiagnosed [3]. The annual HIV incidence is 18 cases per 100,000, and almost half are diagnosed at a late stage [2]. Late HIV diagnosis has a profound individual implication in terms of HIV-related morbidity and mortality [4–6], as well as population implications, since it increases the risk of onward HIV transmission [7,8]. Despite this, several studies have shown that primary care providers frequently miss opportunities to test for HIV [9–11]. Primary care providers are required to perform different preventive care tasks to achieve better quality care, one of which is screening for HIV infection. There is increasing evidence that this testing is acceptable to patients in a primary care environment and is also operationally feasible [12], even though implementation requires training and staff support [13]. There is a need to assess the primary care providers’ knowledge about HIV, and to better understand barriers to HIV testing, with the aim of contributing to a decrease in the number of undiagnosed people [14,15]. To achieve effective control of the HIV epidemic, medical education is necessary, including updated information to primary care providers [16].

The aim of our study is to evaluate the level of knowledge about HIV and to identify any barriers to HIV testing on the part of primary care providers in our healthcare area, using a structured questionnaire. We aim to determine if providers who had ever offered an HIV test exhibited different baseline knowledge and barrier scores, compared to those who had never offered a test. Furthermore, we evaluate whether primary care centers with high HIV testing and new HIV diagnosis rates have providers who obtain better results in the questionnaire. Finally, we aim to assess an education program, to observe whether attendance at a teaching session improves certain variables, including individual knowledge and barriers, and at a primary care center level, HIV testing, new HIV diagnoses, and late diagnosis rates.

## Methods

The DRIVE study (Diagnóstico Rápido de la Infección por VIH en España, or in English ‘Rapid HIV testing in Spain’) was designed to investigate different aspects of HIV testing, and the FOCO study (Formación Concienciación, in English ‘Education Awareness’) was developed with the aim of improving the HIV screening in primary care through the promotion of educational programs. The current analysis, which is part of the FOCO and DRIVE03 studies, was approved by the research Ethics Committee at the Hospital Clínico Universitario of Valencia and Hospital Universitario Ramón y Cajal. It was carried out from January 2016 to December 2017 in 20 primary care centers of the University Hospital Ramón y Cajal: its basic health area has an estimated population of 555,655, with 373,180 aged 15- to 65-years-old.

A cross-sectional study, of baseline anonymously taken questionnaires, was performed by personnel who had attended the training sessions, to assess attitudes towards HIV testing. A self-administered questionnaire developed within the OptTEST project (Project No. 20131102, European Union, Framework of the Health Program 2008–2013), was used [17]. We explored the variables related to primary care centers, age, gender, professional category, along with 16 items regarding knowledge of the field as well as connected barriers, plus three items related to medical practice. Items were categorized as knowledge- or barrier-related, according to an HIV expert committee. This questionnaire was administered at the beginning and end of the training sessions. For the post-training measure, only 14 items were actually used, whereas items about lack of time and difficulty with foreign languages were excluded from the post-intervention questionnaire, as they were not amenable to modification through this training intervention. Each question’s score was calculated with an assigned value for each ordinal answer (1: totally disagree, 5: totally agree), obtaining a result with the sum of individual items. Answers were dichotomized into knowledge, including "present or absent," as well as barriers "present or absent," considering the null response ("neither agree nor disagree") as being inadequate (absent knowledge or present barriers) for specific analyses. Global scores per individual were obtained from a total of 100 points on questions having to do with knowledge and testing barriers.

The training program consisted of a 2-hour session accredited by the Council of the Community of Madrid, which included four modules: (1) epidemiology of HIV infection, (2) diagnostic strategies, (3) treatment of HIV, and (4) other sexually transmitted infections. After the sessions, we had an hour to review the material and complete the questionnaires. The main objective was to promote early diagnosis of HIV infection in the primary care setting by disseminating current HIV testing guidelines; another objective was to improve knowledge of current trends in prevention, diagnosis, and management of HIV infection. In each primary care center, we then analyzed the number of patients who attended, HIV tests performed, and confirmed new HIV diagnoses in the 6 months before and after the training program. We also calculated the HIV testing rate (number of HIV tests performed/estimated number of patients attending) and the new HIV diagnosis rate (new HIV diagnoses/number of tests performed) in this period. Continuous variables of interest, such as HIV testing or new HIV diagnosis rates were categorized as dichotomous for some analyses, using the mean as the cutoff point.

To ascertain the first objective, we compared primary care providers who had offered an HIV test vs. those who do not, using demographic and occupational data obtained through the questionnaire used, as well as items related to depth of knowledge and testing barriers. To evaluate the second objective, the primary care centers were categorized according to their level of HIV testing and new HIV diagnosis rates, with a comparison of the same variables. A quasi-experimental design with pre- and post-intervention measures was performed to investigate the impact of the training program for increased knowledge and reduced barrier scores, HIV testing, new HIV diagnoses, and late diagnosis rates.

Data were stored in an Excel-2011 database (Microsoft, Redmond, WA), using self-developed, automated questionnaire-reading software. Statistical analysis was performed using Stata 15.1 software (Stata Corp-LP, College Station, TX).

For descriptive analyses, we used absolute and relative frequencies, and means and standard deviations, or medians with an interquartile range (IQR). A comparison of categorical variables was performed using the χ^2^ (or Fisher's exact test if necessary), and the Mann-Whitney U test was used for median comparison. The Wilcoxon test determined differences between pairs of observations. Effect sizes were estimated using Cohen’s d, and the confidence interval (CI) 95% was used. We created a binary logistic multivariable model to assess the relationship between variables for the HIV testing offer. All probabilities were two-tailed, and a p value of <0.05 was considered to indicate statistical significance.

## Results

Among 630 primary care providers working in the 20 primary care centers of the Ramón y Cajal basic health area, 454 (72%) attended the training sessions (mean of 23 for each primary care center). Of these, 344 (76%) filled out the initial questionnaire, with women making up 91.7% of respondents, having a median age of 51 years (IQR 43–56). In addition, 59% were medical doctors, 39% were nurses, and 2% were nursing assistants. Of the total who completed at least one questionnaire, 75.1% offered an HIV test in the past (96.7% doctors, 39.5% nurses, and 14.3% nursing assistants), and 98.4% reported acceptance by patients. Moreover, 98% answered that it would be useful to have a tool to identify patients with an indication for HIV testing. The mean score of the question relating to HIV risk assessment in daily practice was 3.18 (3.07–3.29), while only 25% (20–29%) of respondents are aware of national HIV testing guidelines. Lack of time was considered to be one of the least perceived barriers, with a mean score of 1.89 (1.80–1.98), and difficulty with foreign languages was considered to be one of the most perceived barriers, with a mean score of 3.31 (3.20–3.42).

### Profile of primary care providers who offer the HIV test

Table 1 shows population characteristics, as they answer “yes" or "no" to the question "Have you ever offered an HIV test?” Primary care providers who previously offered it had better scores in knowledge-related questions (*p*<0.001) and lower scores in barrier-related questions (p<0.001). Further, 96.7% of medical doctors had offered the test, compared to 39.5% of nursing staff (p<0.001). In the multivariable analysis, after adjusting for professional category, having ever offered an HIV test was independently associated with greater knowledge (p = 0.027) as well as reduced barrier scores (*p* = 0.005). The interaction between professional category and knowledge and barrier scores was fully explored, but found no correlation (p = 0.570 and p = 0.550, respectively).

**Table 1:** Characteristics of providers according to previous offer of an HIV test.

	Previous offer of HIV test		Unadjusted OR (95% CI)	p	Adjusted OR** (95% CI)	p
	Yes (n = 255)	No (n = 85)				
**Age, years, median (IQR)**	50 (44–50)	55 (42–58)	0.99 (0.96–1.01)	0.322	0.99 (0.96–1.02)	0.567
**Female gender (%)**	86.3	87.1	0.98 (0.47–2.04)	0.964	0.99 (0.95–1.03)	0.779
Knowledge score*, median (IQR)	82 (76–87)	73 (68–78)	1.14 (1.10–1.18)	<0.001	1.07 (1.01–1.13)	0.027
Barriers score*, median (IQR)	49 (40–53)	60 (51–67)	0.75 (0.69–0.81)	<0.001	0.89 (0.77–0.95)	0.005
**Professional category (%)**			0.02 (0.01–0.05)	<0.001	**-**	**-**
**Medical doctor**	96.7	3.3				
**Nurse**	39.5	60.5				

*Scores obtained out of 100.

**Adjusting for professional category.

IQR: interquartile range.

Differences by professional category (medicine or nursing) are shown in Table 2, with nursing assistants excluded from the analysis. There were no differences between groups in gender or age, while nurses were more likely to have higher score on barrier items (OR 3.28; CI 95% 2.01–5.46; p<0.001), and reduced scores on knowledge items (OR 0.28; CI 95% 0.17–0.46; p<0.001).

**Table 2:** Population characteristics by professional category.

	Medical doctors (n = 218)	Nurses (n = 119)	p
**Female gender (%)**	85.3	88.2	0.456
**Age, median (IQR)**	50 (45–54)	53 (41–58)	0.809
**Knowledge score, median (IQR)**	82 (76–87)	78 (69–80)	<0.001
**Barrier score, median (IQR)**	49 (40–53)	56 (47–64)	<0.001
**Previous offer of HIV test (%)**	96.7	39.5	<0.001

IQR: interquartile range

### Relationship between primary care centers’ baseline HIV testing, new HIV diagnosis rates, and primary care providers’ knowledge and associated barriers to HIV testing

Table 3 shows differences between primary care centers, categorized according to the HIV testing rate and the new HIV diagnosis rate, using the mean as the cutoff point. There was a marginally significant association between adequate knowledge and centers with increased HIV testing rates (OR 1.51; CI 95% 0.98–2.34; p = 0.063), as well as a statistically significant association between a strong knowledge base and centers with greater new HIV diagnosis rates (OR 1.61; CI 95% 1.04–2.49; p = 0.032). There were no differences in other variables, such as gender, age, professional category, number of staff members, or barrier score.

**Table 3:** Differences between primary care centers, categorized according to their HIV testing rate and new HIV diagnosis rate.

**Variables**	**High HIV testing rate**	**Low HIV testing rate**	**p**
**Female gender (%)**	87.3	85.8	0.685
**Age, median (IQR)**	50 (44–56)	51 (44–56)	0.662
**Professional category**			0.824
** Medical doctors (%)**	63.5	63.2	
** Nurses (%)**	35.4	33.5	
** Nursing assistants (%)**	1.1	3.2	
**Staff number, median (IQR)**	37 (30–45)	29 (23–47)	0.427
**Adequate knowledge (%)**	52.5	42.2	0.063
**Barriers present (%)**	48.9	54.9	0.286
	**High new-HIV diagnosis rate**	**Low new-HIV diagnosis rate**	**p**
**Female gender (%)**	87.3	86.1	0.752
**Age, median (IQR)**	50 (44–56)	50 (43–56)	0.672
**Professional category**			0.144
** Medical doctors (%)**	67.5	59.9	
** Nurses (%)**	30.6	38.0	
** Nursing assistants (%)**	1.9	2.1	
**Staff number, median (IQR)**	35 (33–49)	27 (21–44)	0.073
**Patients attended, median (IQR)**	15621 (13743–19560)	9517 (8603–12507)	0.074
**Adequate knowledge (%)**	54.4	42.5	**0.032**
**Barriers present (%)**	47.5	54.8	0.197

IQR: interquartile range.

### Baseline knowledge and barriers according to the OptTEST questionnaire and the impact of the training program

Before the intervention, we found adequate knowledge levels for most of the questions in the OptTEST questionnaire, with an overall mean score of 84.7 out of 100 points. However, barriers to HIV testing were identified in a large proportion of primary care providers, with an overall score of almost 50 points. Only two of the knowledge-related items were considered “absent knowledge,” with a median score below the cutoff point. They inquired about the need to offer the HIV test, although the patient does not request it (2.04 ± 0.99), and the lack of HIV transmission in patients with an undetectable viral load (1.93 ± 0.91). The main barriers included the difficulty in exploring risk practices and HIV-indicator conditions, which also involves the perceived barrier of a lack of HIV training. The effect of the training program on both knowledge level and barrier scores was assessed in a total of 84 matched questionnaires. The baseline characteristics of the subset of paired OptTEST questionnaires were similar to those of the overall population analyzed, with 91.7% women, a median age of 51 years, with 59% physicians and 39% nurses. Table 4 shows the mean difference for each question before and after the teaching intervention. Statistically significant differences were found in most items (12 out of 14 questions). In this way, the overall mean score in provider’s knowledge was higher (mean difference 6.7 [CI 95% 5.1–8.3], p<0.001), and the barrier score was lower (mean difference 3.9 [CI 95% 1.5–6.2], p = 0.002) after the training intervention. The effect size was larger in knowledge (d = 0.89) than the barrier dimension (d = 0.32).

**Table 4:** Pre- and post-intervention outcomes in knowledge level and barrier scores.

N = 84 matched questionnaires	Pre-intervention score (mean, SD)	Post-intervention score (mean, SD)	p
**KNOWLEDGE**			
**1. People with undiagnosed HIV can be well without any symptoms for years.**	4.70 (0.53)	4.80 (0.56)	0.001
**2. If diagnosed early, HIV can be managed effectively with medication.**	4.69 (0.60)	4.92 (0.28)	0.011
**3. HIV infected people on medication are less likely to transmit the infection.**	4.13 (1.08)	4.69 (0.69)	<0.001
**4. It is important that people know their HIV status.**	4.81 (0.61)	4.93 (0.26)	0.199
**5. HIV testing should only be performed if a patient asks for it.**	2.04 (0.99)	1.69 (0.91)	<0.001
**6. HIV testing should only be offered to people with high risk.**	1.93 (0.91)	1.65 (0.91)	0.007
**7. A leaflet or brief pre-test discussion is sufficient before offering the HIV test.**	3.42 (1.11)	3.80 (1.33)	<0.001
**8. Offering HIV testing to people with indicator conditions is a good idea.**	4.13 (0.91)	4.77 (0.52)	0.003
**Global Knowledge score (out of 100)**	84.7 (7.9)	91.4 (7.3)	<0.001
**BARRIERS**			
**1. I am concerned that patients might ask questions I cannot answer.**	3.01 (1.13)	2.87 (1.21)	<0.001
**2. I prefer that patients ask for testing themselves.**	2.52 (0.83)	2.25 (0.97)	<0.001
**3. I do not think that offering HIV testing will be acceptable to patients.**	1.76 (0.73)	1.57 (0.75)	0.140
**4. I do not have time to include HIV testing as part of patients’ care.**	1.89 (0.83)	-	-
**5. I would require additional training before offering HIV testing.**	3.08 (1.31)	2.95 (1.23)	<0.001
**6. I am comfortable discussing HIV testing with patients.**	3.73 (0.86)	3.94 (0.92)	<0.001
**7. Language barriers prevent some patients from being offered HIV testing.**	3.31 (1.07)	-	-
**8. I am concerned that offering HIV testing will negatively affect patients' opinion about our services.**	1.80 (0.84)	1.63 (0.85)	0.049
**Global Barriers score (out of 100).**	47.8 (11.7)	43.9 (12.9)	0.002

SD: standard deviation.

A median number of 6,226 persons visited every health center in both periods that we studied, 6 months before and after the training program. There were 22 new HIV diagnoses (11 in each period). After finishing the training program, we found a higher HIV testing rate (OR 1.19; CI 1.02–1.42; *p* = 0.036). In addition, we found a trend towards a decrease of advanced HIV disease presentation (OR 0.13; CI 95% 0–1.15; *p* = 0.092), defined as the presence of a CD4 count of <200 cells/μl at diagnosis, although it was not statistically significant. No significant differences were observed, either in the number of new HIV diagnoses or in the new HIV diagnosis rate. We summarize these data in Table 5.

**Table 5:** Pre- and post-intervention outcomes in the 20 primary care centers of the basic health area.

20 primary care centers	Pre-intervention (6 months)	Post-intervention (6 months)	p
**Number of new HIV diagnoses (No.)**	11	11	0.957
**Number of new HIV diagnoses per center, mean (SD)**	0.55 (0.99)	0.55 (0.76)	0.957
**HIV testing rate (%), mean (SD)**	3.68 (1.03)	4.34 (1.37)	**0.036**
**New HIV diagnosis rate (%), mean (SD)**	1.87 (3.20)	2.12 (2.90)	0.790
**CD4, median (IQR)**	381 (133–959)	442 (339–489)	0.772
**CD4%, median (IQR)**	21.9 (9.4–40.8)	28.0 (17.0–37.0)	0.677
**Late diagnosis, No. (%)**			
** <350 CD4+**	4 (36.4)	3 (27.3)	0.227
** <200 CD4+**	4 (36.4)	1 (9.1)	0.092

IQR: interquartile range; SD: standard deviation.

## Discussion

This study highlights how rates of HIV testing and new diagnoses are influenced by providers' knowledge about the disease itself and the barriers in testing it. Furthermore, it demonstrates the possibility of modifying knowledge and reducing perceived barriers through educational programs. This research was carried out in the Ramón y Cajal basic health area, with broad participation of the total primary care staff, which sees adequate levels of knowledge in most items of the OptTEST questionnaire, yet some barriers to HIV testing are still seen in this setting. Almost all respondents report good acceptance after offering the HIV test, similar to other studies in primary care [18]; this is more positive than indicated in other settings, such as emergency services [19]. Despite this, low HIV testing rates are still seen in primary care centers of this health area [20]

We question knowledge-related parameters, in which there could be more improvement, about indications for HIV testing and HIV transmissibility in patients with an undetectable viral load. This should instigate reflection about the need to implement other measures, while taking into account that more than half of the total new HIV cases are diagnosed at primary care centers in this health area [21].

When examining barriers to testing, our study shows that primary care staff are aware of the need for more training on HIV to address the fear of patient questions; this is also to encourage enquiring about possible risk behaviors and considering HIV-indicator conditions in daily practice. Previous data suggest that primary care providers are inconsistent in their practice in relation to screening and identification of HIV-risk behaviors during brief consultations. Systematic screening for HIV risk would reduce variability and increase detection of risk behaviors [22]. Other work indicates structural reasons, e.g., lack of time to explain being the main barriers to screening [15]. However, we do not find this barrier in our health area, with less than 20% of staff indicating it is a concern.

Global scores of the OptTEST questionnaire are useful in evaluating the effect of any intervention that promotes HIV testing; we mainly distinguish between primary care providers who had ever offered an HIV test from those who did not. The lack of experience and training is the main concern among respondents who had not previously tested patients for HIV: this underlines the importance of developing targeted education programs. This has also been identified in previous work, in which respondents perceive that necessary financial resources are already available, but that implementation of educational strategies could be helpful as well [23]. Periodic training of primary care providers is essential; they work in a strategic setting in the health system, and their mission is to carry out tasks for both health promotion and prevention: this would foster early HIV diagnosis in a large percentage of people living with undiagnosed HIV infection. Several studies are now being conducted on the development of education strategies, with the objective of improving HIV prevention with the use of pre-exposure prophylaxis (PrEP) [24,25], although few studies are focused on primary care. Some educational interventions in other settings, such as Sexual Health in Practice (SHIP) in the UK [26], or the US network of AIDS Education and Training Centers (AETCs) [27], demonstrate a significant and sustained increase in HIV testing on the part of primary care providers in the absence of financial incentives. This training addresses barriers to HIV testing as expressed by participants, with a strong indication for strategies to overcome them. We must address providers’ perceptions, related to the local context, to promote new, routine clinical practices [28].

Nearly all primary care providers who complete the questionnaire considered it useful to have a tool to identify patients with an indication for HIV testing, which would overcome some current barriers. Different tools are being developed for this purpose, such as the creation of questionnaires to evaluate risk behaviors and HIV-indicator conditions [29–31]; this would facilitate a decision about the test offer. Exhaustive directed screening might achieve similar or better HIV diagnosis rates than non-directed or universal screening; it is more focused, and uses fewer resources than universal screening while maintaining a predictive negative value of 100% [32].

In our study, 39.5% of nurses requested an HIV test at some point in their careers, which is a lower percentage than medical doctors (96.7%, *p*<0.001). In addition, they scored lower in knowledge-related items and higher in barrier-related items (*p*<0.001) than physicians. The nursing staff are a professional group with high level of contact with patients, and have a key role, not only in terms of delivering care for people living with HIV, but in prevention and diagnosis, detecting risk behaviors, and carrying out screening protocols. In our setting, some studies show the role of HIV specialist nurses [33], although adequate information on their role in the primary care population is not available. Attitudes towards HIV screening have been studied in the nursing staff, with the aim of understanding variables that influence them. This is essential to establishing measures to change negative attitudes and promote better health care [34]. The need to implement measures for this population is evident, which would improve their understanding about the infection and barriers to screening.

There are several limitations to this study. The effect of the intervention was not evaluated by a randomized controlled trial. There was good participation of primary care providers, even though it did not reach 100%. This could lead to a selection bias; therefore, the paired responses obtained could correspond to individuals with greater motivation. The effect of the education program was measured 6 months post-intervention; it is thus necessary to conduct studies to assess long-term effects. The low prevalence of HIV in our area could affect the analysis, but training strategies may also be of value in lower prevalence areas, as late HIV diagnosis is more common [35]. We should deliver these strategies, but it is not yet clear what the best implementation would be in clinical practice. We do not have enough evidence to know if training will be sufficient, as our results only show a moderate reduction in those diagnosed with a CD4 count of <200 cells/μl and no difference in the number of new HIV diagnoses (or the new HIV diagnosis rate). Other interventions could reduce late diagnoses and increase the number of new HIV diagnoses. Further investigation into educational interventions with patient-oriented programs is required to understand long-term effects, and whether they can impact late HIV-infection diagnosis.

Three of the main findings of this work point in the same direction. First, as previously discussed, primary care providers who had offered an HIV test show significantly higher knowledge and lower barriers scores, regardless of their professional category. Second, there is an association between primary care centers with higher scores on knowledge-related questions and increased HIV testing, and new HIV diagnosis rates. Finally, we look at how training intervention improves global knowledge and barrier scores. This highlights the relevance of training in reducing low HIV testing rates, current high late diagnosis rates, and in the search for more effective ways to combat the HIV epidemic.

Our data support the conclusion that educational programs can lead to changes in primary care providers’ behavior. These programs are crucial to achieve enhanced screening and new-HIV diagnosis rates, and reduce undiagnosed HIV infection.
